# The Seasonal Microbial Ecology of Plankton and Plankton-Associated Vibrio parahaemolyticus in the Northeast United States

**DOI:** 10.1128/AEM.02973-20

**Published:** 2021-07-13

**Authors:** Meghan A. Hartwick, Audrey Berenson, Cheryl A. Whistler, Elena N. Naumova, Stephen H. Jones

**Affiliations:** a Northeast Center for Vibrio Disease and Ecology, University of New Hampshire, Durham, New Hampshire, USA; b Department of Molecular, Cellular, and Biomedical Sciences, University of New Hampshire, Durham, New Hampshire, USA; c Department of Natural Resources and the Environment, University of New Hampshire, Durham, New Hampshire, USA; d Division of Nutrition Data Sciences, Friedman School of Nutrition Science and Policy, Tufts University, Boston, Massachusetts, USA; The Pennsylvania State University

**Keywords:** climate change, plankton, public health, seafood safety, seasonality, vibrio

## Abstract

Microbial ecology studies have proven to be important resources for improving infectious disease response and outbreak prevention. Vibrio parahaemolyticus is an ongoing source of shellfish-borne food illness in the Northeast United States, and there is keen interest in understanding the environmental conditions that coincide with V. parahaemolyticus disease risk, in order to aid harvest management and prevent further illness. Zooplankton and chitinous phytoplankton are associated with V. parahaemolyticus dynamics elsewhere; however, this relationship is undetermined for the Great Bay estuary (GBE), an important emerging shellfish growing region in the Northeast United States. A comprehensive evaluation of the microbial ecology of V. parahaemolyticus associated with plankton was conducted in the GBE using 3 years of data regarding plankton community, nutrient concentration, water quality, and V. parahaemolyticus concentration in plankton. The concentrations of V. parahaemolyticus associated with plankton were highly seasonal, and the highest concentrations of V. parahaemolyticus cultured from zooplankton occurred approximately 1 month before the highest concentrations of V. parahaemolyticus from phytoplankton. The two V. parahaemolyticus peaks corresponded with different water quality variables and a few highly seasonal plankton taxa. Importantly, V. parahaemolyticus concentrations and plankton community dynamics were poorly associated with nutrient concentrations and chlorophyll *a*, commonly applied proxy variables for assessing ecological health risks and human health risks from harmful plankton and V. parahaemolyticus elsewhere. Together, these statistical associations (or lack thereof) provide valuable insights to characterize the plankton-V. parahaemolyticus dynamic and inform approaches for understanding the potential contribution of plankton to human health risks from V. parahaemolyticus for the Northeast United States.

**IMPORTANCE** The *Vibrio*-plankton interaction is a focal relationship in *Vibrio* disease research; however, little is known about this dynamic in the Northeast United States, where V. parahaemolyticus is an established public health issue. We integrated phototactic plankton separation with seasonality analysis to determine the dynamics of the plankton community, water quality, and V. parahaemolyticus concentrations. Distinct bimodal peaks in the seasonal timing of V. parahaemolyticus abundance from phyto- versus zooplankton and differing associations with water quality variables and plankton taxa indicate that monitoring and forecasting approaches should consider the source of exposure when designing predictive methods for V. parahaemolyticus. Helicotheca tamensis has not been previously reported in the GBE. Its detection during this study provides evidence of the changes occurring in the ecology of regional estuaries and potential mechanisms for changes in V. parahaemolyticus populations. The *Vibrio* monitoring approaches can be translated to aid other areas facing similar public health challenges.

## INTRODUCTION

The ecology of Vibrio parahaemolyticus has been the focus of numerous intensive studies due to its role as a human and animal pathogen (1–5). A wide array of environmental variables has been reported in association with V. parahaemolyticus dynamics, including water temperature, salinity, pH, inorganic and organic nutrients, suspended solids, turbidity, chlorophyll *a*, light availability, and meteorological conditions (6–13). Kaneko and Colwell (14) were the first to suggest that plankton could contribute to V. parahaemolyticus dynamics in the Chesapeake Bay as a key source of nutrients for growth and persistence and also provide protection from predation. More recent studies in the United States from the Chesapeake Bay and other shellfish regions, such as Delaware, Mississippi, North Carolina, and Washington State, have also demonstrated associations between V. parahaemolyticus and plankton (or plankton-associated variables such as chlorophyll *a*) that vary in strength (2, 9, 15–17).

**FIG 1:**
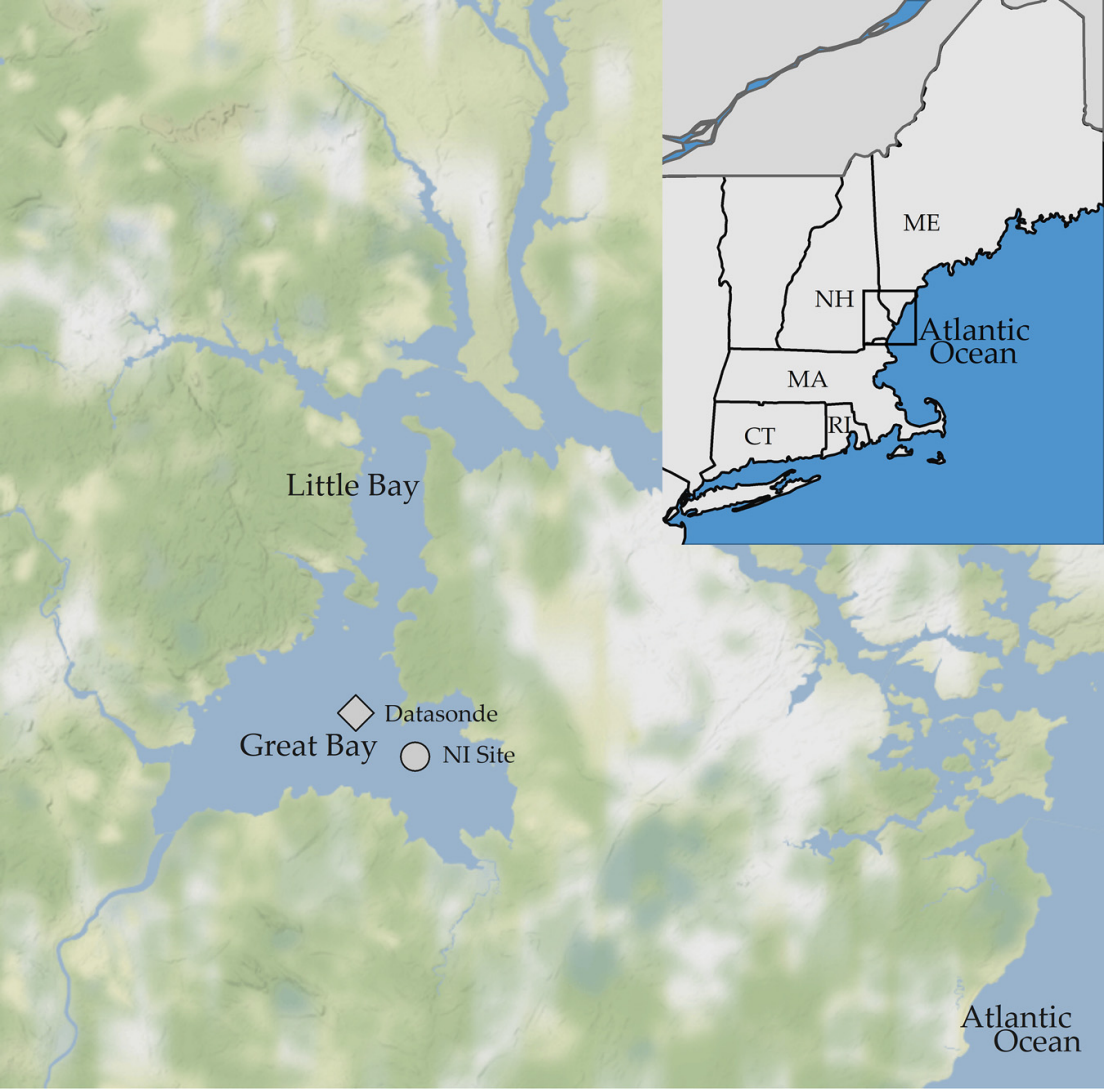
The Great Bay estuary. The Nannie Island study site is indicated by the circle, and the datasonde is shown with the diamond. (Maps created with ggmaps.)

Both V. parahaemolyticus disease and harmful algal blooms are increasing worldwide, concurrent with climate-related changes in the marine environment, and increasing V. parahaemolyticus disease could in part be driven by the altered plankton dynamics (3, 18–21). The changes in the global V. parahaemolyticus community are also observed locally in the Great Bay estuary (GBE) in the Northeast United States (Fig. 1). V. parahaemolyticus concentrations have become more variable, with higher peak concentrations throughout summer, and remain high late into fall months, when previously concentrations would have been at or near detection limits (6, 12).

Previous modeling efforts to characterize these changes in observed V. parahaemolyticus dynamics included measured nutrients and chlorophyll *a* as proxy variables for plankton. However, the outcomes of these studies were mixed as they related to the role of plankton in V. parahaemolyticus ecology (6, 8, 12). We hypothesized that a direct study of the plankton community and associated V. parahaemolyticus dynamics in the GBE would yield a more comprehensive and informative understanding of how plankton may affect V. parahaemolyticus in this region.

Little is currently known about plankton community dynamics in the GBE, and this type of study has not been conducted previously. Therefore, the goals of this work were (i) to identify the taxa in the plankton community, (ii) to determine whether individual taxa or overall plankton concentrations covaried with V. parahaemolyticus concentrations, and (iii) to identify water quality variables that covaried with plankton and associated V. parahaemolyticus in order to improve V. parahaemolyticus modeling and risk forecasting in this region. This study is the first comprehensive, multiyear assessment of plankton and concentrations of V. parahaemolyticus from plankton in the GBE and the Northeast United States.

## RESULTS

Sampling began in July 2014, while in 2015 and 2016, sampling began when small-craft vessels could be safely operated dependent on ice-out and seasonal conditions in March (2016) and May (2015). Sampling ended for the same reason in November (2015) or December (2014 and 2016). Thirty-one total sampling events with complete data for V. parahaemolyticus concentrations, plankton community analysis, water quality, and nutrients were conducted. V. parahaemolyticus concentrations and plankton total abundance varied widely overall (Table 1); however, they were consistent between years (see Table S1 in the supplemental material). Water temperature, salinity, pH, turbidity, and chlorophyll *a* and total dissolved nitrogen (TDN) concentrations were consistent with data in previous reports (6, 8, 22).

**TABLE 1:** Ranges and mean values for V. parahaemolyticus, water quality, and nutrients

Variable^*a*^	Min	Max	Mean ± SD
*Vp* and plankton			
Phytoplankton *Vp* (MPN/liter)	0.018	14	1.7 ± 4
Zooplankton *Vp* (MPN/liter)	0.018	21	8.5 ± 2.6
Total plankton abundance (no./liter)	83	35,853	6,700 ± 11,229
Phytoplankton abundance (no./liter)	62	35,630	6,474 ± 10,980
Zooplankton abundance (no./liter)	0	3,350	227 ± 381
Water quality			
DON (mg/liter)	0.0	0.2	0.12 ± 0.05
NH_4_ (mg N/liter)	0.0	0.1	0.02 ± 0.02
NO_3_ + NO_2_ (mg N/liter)	0.01	0.2	0.04 ± 0.04
NPOC (mg/liter)	0.36	3.8	2.31 ± 0.7
PC (mg/liter)	0.43	3.6	1.16 ± 0.7
PN (mg/liter)	0.05	0.5	0.17 ± 0.1
PO_4_ (mg P/liter)	0.00	0.07	0.03 ± 0.02
TDN (mg/liter)	0.06	0.34	0.18 ± 0.06
Chlorophyll *a* (μg/liter)	1.3	22.6	6.3 ± 4.5
Dissolved oxygen (mg/liter)	6.5	11.5	8.5 ± 1.3
pH	7.5	8.0	7.8 ± 0.15
Pheophytin (μg/liter)	0.7	9.8	2.9 ± 2.2
Salinity (ppt)	14.1	32.1	27.1 ± 3.5
Water temp (°C)	6.3	25.3	17.7 ± 5.1
Total suspended solids (mg/liter)	10.7	76.4	29.8 ± 15.6
Turbidity (NTU)	1.15	163.4	10.8 ± 13.3

a *Vp*, *V. parahaemolyticus*; NTU, nephelometric turbidity units.

### Vibrio parahaemolyticus and plankton abundance.

**FIG 2:**
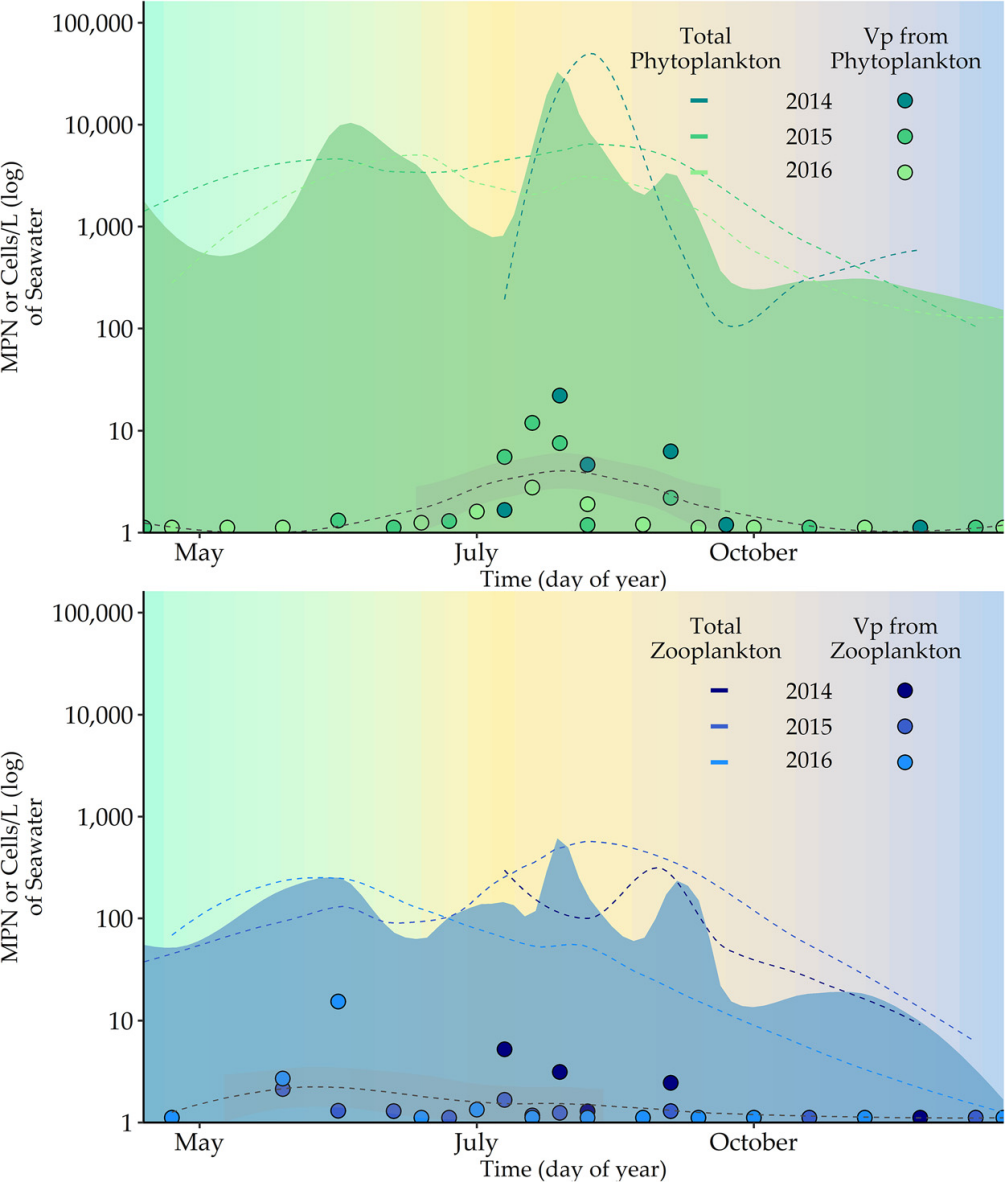
(Top) Total phytoplankton and V. parahaemolyticus from phytoplankton and (bottom) total zooplankton and V. parahaemolyticus from zooplankton superimposed by time for 3 years. Each dotted line is a LOESS (locally estimated scatterplot smoothing) smoother, and the shaded area represents a 95% confidence interval. Cooler spring and fall months are shown in blue and green. Warmer summer months are in yellow to red.

Plankton were present in every sample throughout the study period. However, V. parahaemolyticus (detection limit is a most probable number [MPN] of >0.03/g) was detected in only 54.8% of phytoplankton samples (17/31) and 45.1% of zooplankton samples (14/31). Phytoplankton concentrations (cells/liter) (Fig. 2, shown in green) were highest during the spring bloom and in later summer months. V. parahaemolyticus concentrations from phytoplankton were also elevated during the summer months, but unlike phytoplankton concentrations, this organism was not detected in spring and late fall months.

Zooplankton (Fig. 2, shown in blue) were present in the water column year-round but at low levels during spring and fall. V. parahaemolyticus was not detected in zooplankton samples during cooler months, when zooplankton levels in the water column declined.

Thirty-four individual taxa were identified and classified into the following groups: diatoms, dinoflagellates, zooplankton, and “other” (one Haptophyte and one Chrysophyte) (Table 2). Diatoms were most abundant (relative abundance [RA] = 96.4%) followed by zooplankton (RA = 2.8%) and dinoflagellates (RA = 1.1%). The diatoms *Chaetoceros* spp., Helicotheca tamensis, *Navicula* spp., and *Skeletonema* spp. were classified as abundant, as they had the highest total and relative abundance, accounting for 89.8% of the total abundance over the entire study period. *Fragilariopsis* spp. and *Navicula* spp. were present in all 31 samples. The majority (77.8%) of phyto- and zooplankton taxa sampled during this 3-year period had been previously identified in the GBE (Table 2) (23, 24).

**TABLE 2:** Overall taxa, type, abundance, and frequency of phytoplankton and zooplankton (>53 μm) calculated from all 31 samples from the GBE

Plankton	Type	Observed historically^*a*^	Annual^*b*^	Classification	Total abundance (no. of cells)	Relative abundance (%)	Frequency^*c*^
*Chaetoceros* spp.	Diatom	Yes	Yes	Abundant	145,262	58.6	0.81
*Navicula* spp.	Diatom	Yes	Yes	Abundant	36,102	14.6	1.00
*Helicotheca tamensis*	Diatom	No	Yes	Abundant	22,098	8.9	0.89
*Skeletonema* spp.	Diatom	Yes	Yes	Abundant	19,192	7.7	0.53
*Tintinnida*	Zooplankton	Yes	Yes	Common	4,946	2.0	0.65
*Fragilariopsis* spp.	Diatom	Yes	Yes	Common	4,385	1.8	1.00
Nauplii	Zooplankton	Yes	Yes	Common	2,662	1.1	0.89
*Coscinodiscus* spp.	Diatom	Yes	Yes	Common	2,396	1.0	0.86
*Pleurosigma* spp.	Diatom	No	Yes	Common	2,020	0.8	0.78
*Thalassiosira* spp.	Diatom	Yes	Yes	Common	1,596	0.6	0.20
*Thalassionema* spp.	Diatom	Yes	Yes	Common	1,347	0.5	0.27
*Cylindrotheca* spp.	Diatom	Yes	Yes	Common	896	0.4	0.51
*Licmophora* spp.	Diatom	Yes	Yes	Common	846	0.3	0.54
*Rhizosolenia* spp.	Diatom	Yes	Yes	Common	833	0.3	0.57
*Copepods*	Zooplankton	Yes	Yes	Common	792	0.3	0.78
*Stephanopyxis* spp.	Diatom	No	Yes	Common	531	0.2	0.35
*Bacillaria* spp.	Diatom	Yes	Yes	Common	450	0.2	0.48
*Biddulphia* spp.	Diatom	Yes	Yes	Common	403	0.2	0.48
*Ditylum* spp.	Diatom	Yes	No	Common	274	0.1	0.24
*Grammatophora* spp.	Diatom	Yes	Yes	Rare	141	0.06	0.19
*Leptocylindrus danicus*	Diatom	No	Yes	Rare	112	0.05	0.02
*Odontella* spp.	Diatom	No	No	Rare	100	0.04	0.13
*Ceratium* spp.	Dinoflagellate	Yes	Yes	Rare	72	0.03	0.11
*Detonula* spp.	Diatom	Yes	No	Rare	51	0.02	0.19
*Phaeocystis*	Other	Yes	No	Rare	50	0.02	0.11
*Pseudo-nitzschia* spp.	Diatom	Yes	No	Rare	48	0.02	0.05
*Dinobryon* spp.	Other	Yes	No	Rare	28	0.01	0.11
*Eucampia* spp.	Diatom	Yes	No	Rare	17	0.01	0.03
*Asterionellopsis* spp.	Diatom	Yes	No	Rare	14	0.01	0.11
*Prorocentrum* spp.	Dinoflagellate	Yes	No	Rare	13	0.01	0.03
*Gonyaulax* spp.	Dinoflagellate	No	No	Rare	11	<0.01	0.03
*Corethron* spp.	Diatom	Yes	No	Rare	3	<0.01	0.03
*Gymnodinium* spp.	Dinoflagellate	Yes	No	Rare	3	<0.01	0.3
Cladocerans	Zooplankton	Yes	Yes	Rare	3	<0.01	0.01

a Categorizes whether the specified taxon has been documented in the GBE in previous studies.

b The taxon was detected in multiple years of the study.

c Frequency of detection (from 31 samples) for each taxon.

### Seasonality in the plankton community, Vibrio parahaemolyticus, and water quality variables.

**FIG 3:**
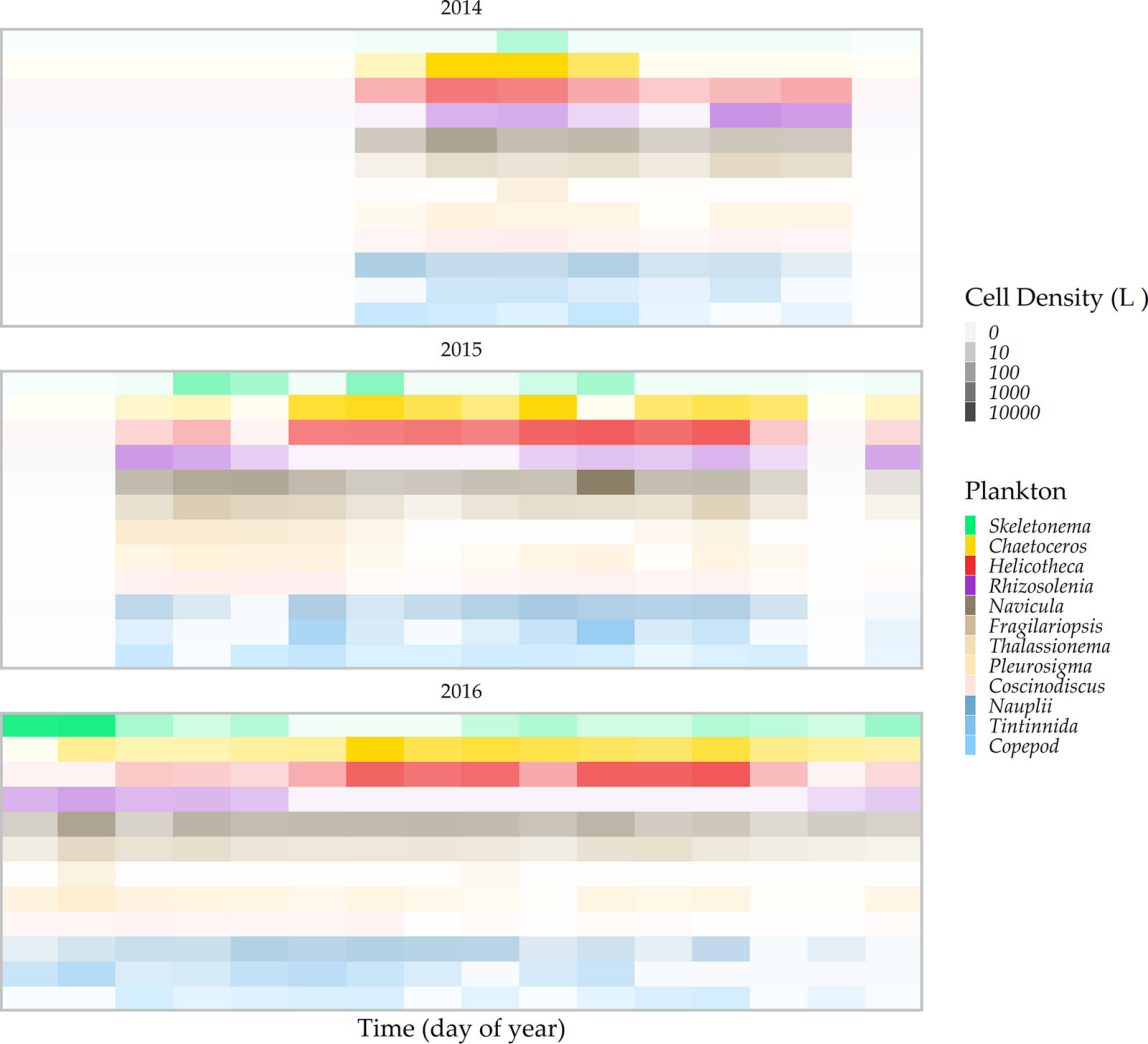
Individual plankton taxon abundance ordered by time in (top) 2014, (middle) 2015, and (bottom) 2016. Plankton taxa in red and yellow were most abundant in warm months and taxa in blue or green were more abundant in cooler spring or fall months.

Each plankton sample contained an average of 14 unique phyto- and zooplankton taxa (minimum = 6; maximum = 21) (Fig. 3). *Chaetoceros* spp. and *Helicotheca tamensis* were present at high concentrations and dominated the plankton community during summer months (Shannon diversity index [H] < 2). Samples from the spring and fall months were generally more diverse (Shannon H > 2), with the exception of early spring samples, which were predominantly *Skeletonema* spp. For example, in 2016, *Skeletonema* sp. concentrations exceeded 7,700 cells/liter in three samples in the spring months, and these samples were comparatively less diverse than other samples from spring months. Nauplii and *Navicula* spp. were generally present at low levels throughout the study period, except in July 2015, when both were detected at >1,000 cells/liter.

The plankton community was strongly seasonal, and *Skeletonema* spp., *Chaetoceros* spp. and *Helicotheca tamensis*, and *Rhizosolenia* spp. were differentiated by indicator species analysis (ISA) into distinct spring, summer, and fall assemblages, respectively (Table 3). These taxa were detected at approximately the same time in each year of the study and in association with other taxa that appeared in spring, summer, and fall months. Only *Coscinodiscus* spp. and *Thalassionema* spp. were not detected in multiple years of the study, as they were detected sporadically in 2015 and 2016. This was identified in our multiresponse permutation procedure (MRPP) analysis but did not alter the season-specific outcomes.

**TABLE 3:** Multivariate analysis of the plankton community composition compared between seasons and years

Group	MRPP	Indicator species^*a*^
Season		
Summer vs fall and spring	0.003	*Chaetoceros*,** Helicotheca**
Summer vs spring	0.003	*Skeletonema*,** Biddulphia,** Stephanopyxis**
Fall vs spring	0.006	Rhizosolenia**
Yr		
2014 vs 2015	0.882	0
2014 vs 2016	0.039	Coscinodiscus**
2015 vs 2016	0.048	*Coscinodiscus*,** Thalassionema**

a Significance of coefficients is indicated as follows: ***, *P* < 0.001; **, *P* < 0.01; *, *P* < 0.1.

The season-specific patterns of the plankton community were also identified by seasonality models 1 and 2 (Table 4) in individual taxa but were less pronounced in the overall abundance of phyto- and zooplankton. *Helicotheca tamensis* (0.63 variance explained), followed by *Rhizosolenia* spp. (0.55 variance explained), *Chaetoceros* spp. (0.39 variance explained), and *Skeletonema* spp. (0.35 variance explained), was the most strongly seasonal and best fit with harmonic regression (model 2). The estimated peak timing of the summer-specific *Helicotheca tamensis* and *Chaetoceros* spp. occurred in August around day 223 ± 11 and day 217 ± 18, respectively. The seasonality of V. parahaemolyticus concentrations from phytoplankton were also well fit by the harmonic regression in model 2, and peak timing was estimated to occur in August at day 224 ± 16.

**TABLE 4:** Variables that demonstrate significant seasonality based on photoperiod and harmonic regression modeling

Variable^*a*^	Coefficient^*b*^		SE		*r*^2^	Deviance explained	AIC	Peak timing (day)^*c*^
	Trend	Seasonality	Trend	Seasonality				
*Vp* from phytoplankton	−0.003	0.29	0.001	0.21	0.20	0.24	156.89	
	−0.002**	−1.89***, −2.16***	0.001	0.43, 0.54	0.53	0.58	138.7	224 ± 16
*Vp* from zooplankton	−0.003**	0.45**	0.001	0.16	0.36	0.40	130.86	
	−0.002**	−0.14***, −1.55***	0.001	0.47, 0.55	0.35	0.41	132.36	188 ± 34
Total plankton abundance	<0.001	0.43	<0.001	0.15	0.15	0.20	147.29	
	<0.001	−0.09, −1.54**	0.001	0.44, 0.53	0.15	0.22	148.33	186 ± 32
Phytoplankton abundance	<0.001	0.41*	0.001	0.16	0.11	0.16	151.87	
	<0.001	−0.12, −1.48**	0.001	0.47, 0.57	0.11	0.18	152.98	187 ± 35
Zooplankton abundance	<0.001	0.53***	0.001	0.13	0.32	0.35	135.59	
	<0.001	0.01, −1.79***	0.001	0.37, 0.45	0.32	0.37	136.67	182 ± 24

*Helicotheca tamensis*	<0.001	<0.001	<0.001	<0.001	0.05	0.10	176.70	
	0.002*	−2.7***, −3.3***	0.001	0.5, 0.5	0.59	0.63	150.1	223 ± 11
*Rhizosolenia* spp.	−0.002	−0.33**	0.001	0.16	0.11	0.16	141.93	
	−0.003***	1.5***, 2.1***	0.001	0.3, 0.4	0.51	0.55	127.2	221 ± 14
*Chaetoceros* spp.	0.002	0.53	0.002	0.31	0.04	0.09	191.63	
	0.004**	−2.4***, −3.4***	0.002	0.7, 0.9	0.34	0.39	185.1	217 ± 18
*Copepod*	−0.002*	0.22	<0.001	0.12	0.2	0.28	124.84	
	−0.001*	−0.32, −0.92*	<0.001	0.34, 0.42	0.25	0.31	124.96	202 ± 36
Nauplii	−0.001	0.51***	<0.001	0.14	0.27	0.32	136.92	
	−0.001	−0.5**, −1.9***	0.001	0.3, 0.5	0.34	0.39	137.1	196 ± 20
*Ditylum* spp.	0.002**	0.24	<0.001	0.12	0.18	0.23	122.02	
	0.002**	−0.11, 0.88*	<0.001	0.33, 0.41	0.18	0.25	123.26	190 ± 40
*Navicula* spp.	−0.002	0.39***	<0.001	0.14	0.15	0.20	134.43	
	−0.002	0.20, −1.21*	<0.001	0.39, 0.48	0.13	0.21	136.09	173 ± 39
*Biddulphia* spp.	<0.001	0.28*	<0.001	<0.001	0.14	0.09	137.79	
	−0.001	1.12**, −0.27	<0.001	0.38, 0.46	0.19	0.26	132.66	105 ± 48
*Coscinodiscus* spp.	−0.003***	0.24	<0.001	0.13	0.4	0.44	128.20	
	−0.004***	1.00**, −0.24	<0.001	0.33, 0.41	0.5	0.55	122.62	105 ± 47
*Thalassionema* spp.	−0.002	0.27	0.001	0.18	0.08	0.13	152.77	
	−0.002*	1.18**, −0.19	0.001	0.48, 0.59	0.17	0.24	149.66	101 ± 58
*Pleurosigma* spp.	0.003	0.19	0.001	0.18	0.02	0.04	150.79	
	−0.0003	1.37**, 0.14	0.001	0.44, 0.54	0.17	0.24	144.21	85 ± 43
*Skeletonema* spp.	0.003	0.03	0.001	0.22	0.03	0.09	167.11	
	0.002	1.8***, 0.9***	0.001	0.5, 0.3	0.29	0.35	160.8	64 ± 31
*Stephanopyxis* spp.	−0.002	−0.08	0.001	0.16	0.01	0.07	143.75	
	−0.03	2.7**, 1.5	0.14	0.84, 1.04	0.20	0.27	156.43	62 ± 32

PO_4_	<0.001	<0.001	<0.001	<0.001	0.05	0.03	161.06	
	<0.001	−0.007***, −0.008*	<0.001	0.003, 0.003	0.63	0.66	199.33	253 ± 16
Salinity	0.004	−0.33	0.002	0.37	0.05	0.11	179.22	
	0.005**	−4.6***, −2.2***	0.002	0.5, 0.7	0.76	0.78	134.87	248 ± 2
Dissolved oxygen	<0.001	−0.18	<0.001	0.13	0.02	0.08	112.91	
	<0.001	1.7***, 1.8***	<0.001	0.03, 0.04	0.83	0.84	56.42	227 ± 7
Water temp	<0.001	1.65	0.002	0.47	0.27	0.32	195.15	
	<0.001	−5.8***, −9.7***	0.0002	0.37, 0.49	0.94	0.95	115.86	213 ± 2
NO_3_ + NO_2_	<0.001	0.01***	<0.001	<0.001	0.37	0.41	119.35	
	<0.001	0.05*, 0.07***	0.008	0.009, 0.04	0.47	0.52	126.39	200 ± 14

a For each variable, the first row shows data for model 1 and the second row shows data for model 2, for sine and cosine terms. *Vp*, V. parahaemolyticus.

b Significance of coefficients is indicated as follows: ***, *P* < 0.001; **, *P* < 0.01; *, *P* < 0.1.

c Peak timing (day of year) estimates are presented as means and standard errors; for two parameters (DO and TDN), the estimates reflect the seasonal nadir.

Nauplii and zooplankton-associated V. parahaemolyticus were well fit by model 1 and 2, and peak timing was similar (around day 188 ± 34 and 182 ± 24, respectively). Taxa that were infrequently detected and at low abundance were not well fit by the seasonal variables in model 1 or 2 (see Table S2).

The variables water temperature, salinity, dissolved oxygen (DO), and PO_4_ were best fit by harmonic regression in model 2 (>0.65 deviance explained) and peak timing occurred after day 213. NO_3_ + NO_2_ was well fit by models 1 and 2, with peak timing around day 200 ± 14. Chlorophyll *a*, pheophytin, turbidity, and the other measured nutrients had little or no seasonality based on poor fits to both models (Table S1; Fig. S2).

### Correlation analysis.

**FIG 4:**
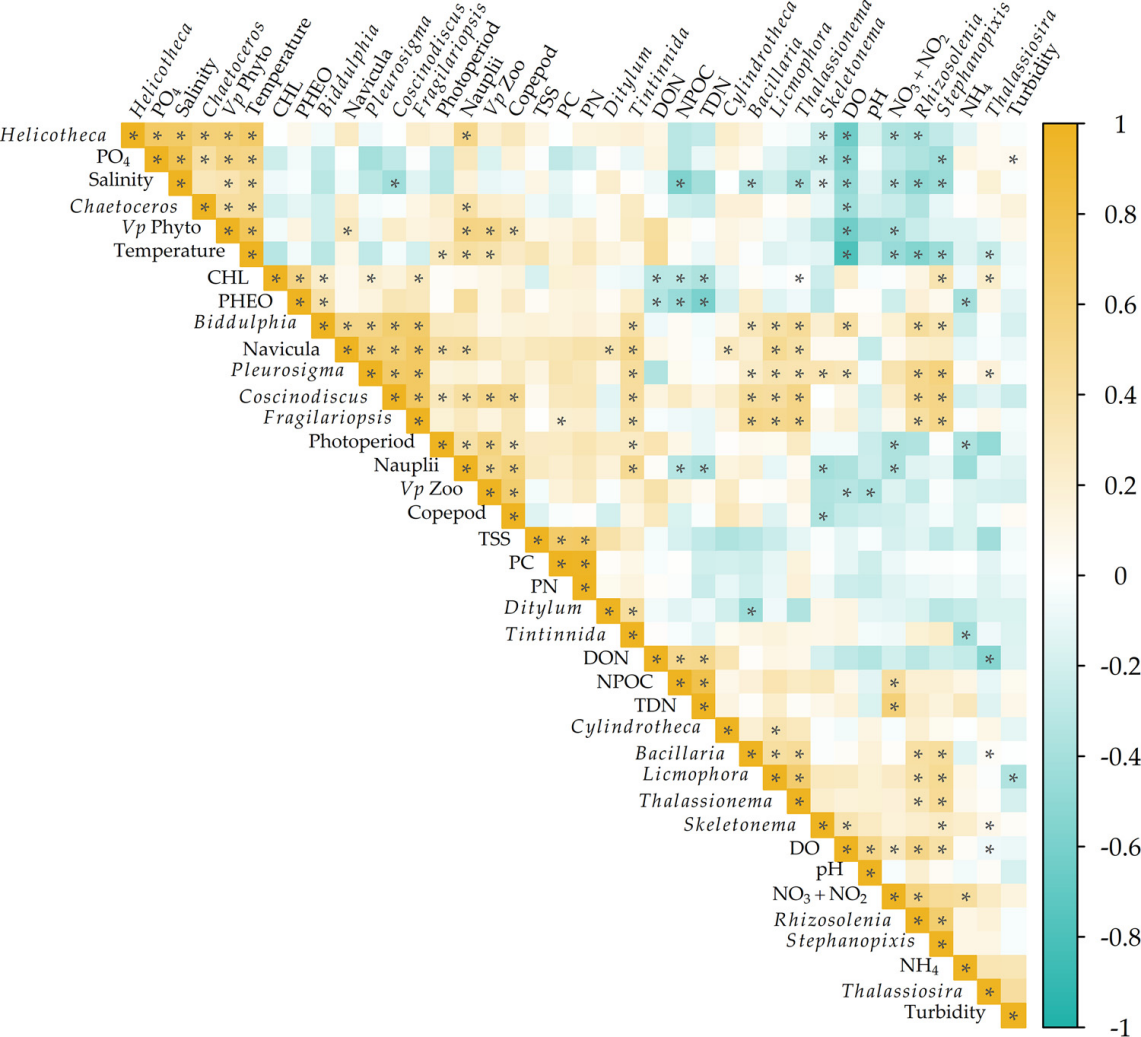
Spearman correlation analysis of V. parahaemolyticus in phyto- and zooplankton, plankton taxa, water quality, and nutrients. Asterisks indicate significant correlations.

A cluster of significant positive correlations between V. parahaemolyticus concentrations in phytoplankton, *Helicotheca tamensis*, *Chaetoceros* spp., PO_4_, salinity, and water temperature is present in the upper left corner of Fig. 4. Likewise, clustering and significant correlative relationships between V. parahaemolyticus concentrations in zooplankton, photoperiod, nauplii, and copepods are also present in the upper middle section of Fig. 4. DO, NO_3_ + NO_2_, *Rhizosolenia* spp., and *Stephanopyxis* spp. produced a separate significant cluster but were negatively correlated with variables that clustered with V. parahaemolyticus concentrations in phyto- or zooplankton. No significant correlations were identified between chlorophyll *a*, pheophytin, total suspended solids (TSS), particulate nitrogen (PN), and particulate carbon (PC) and the majority of individual plankton taxa or V. parahaemolyticus concentrations.

### Seasonal microbial ecology in the GBE.

**FIG 5:**
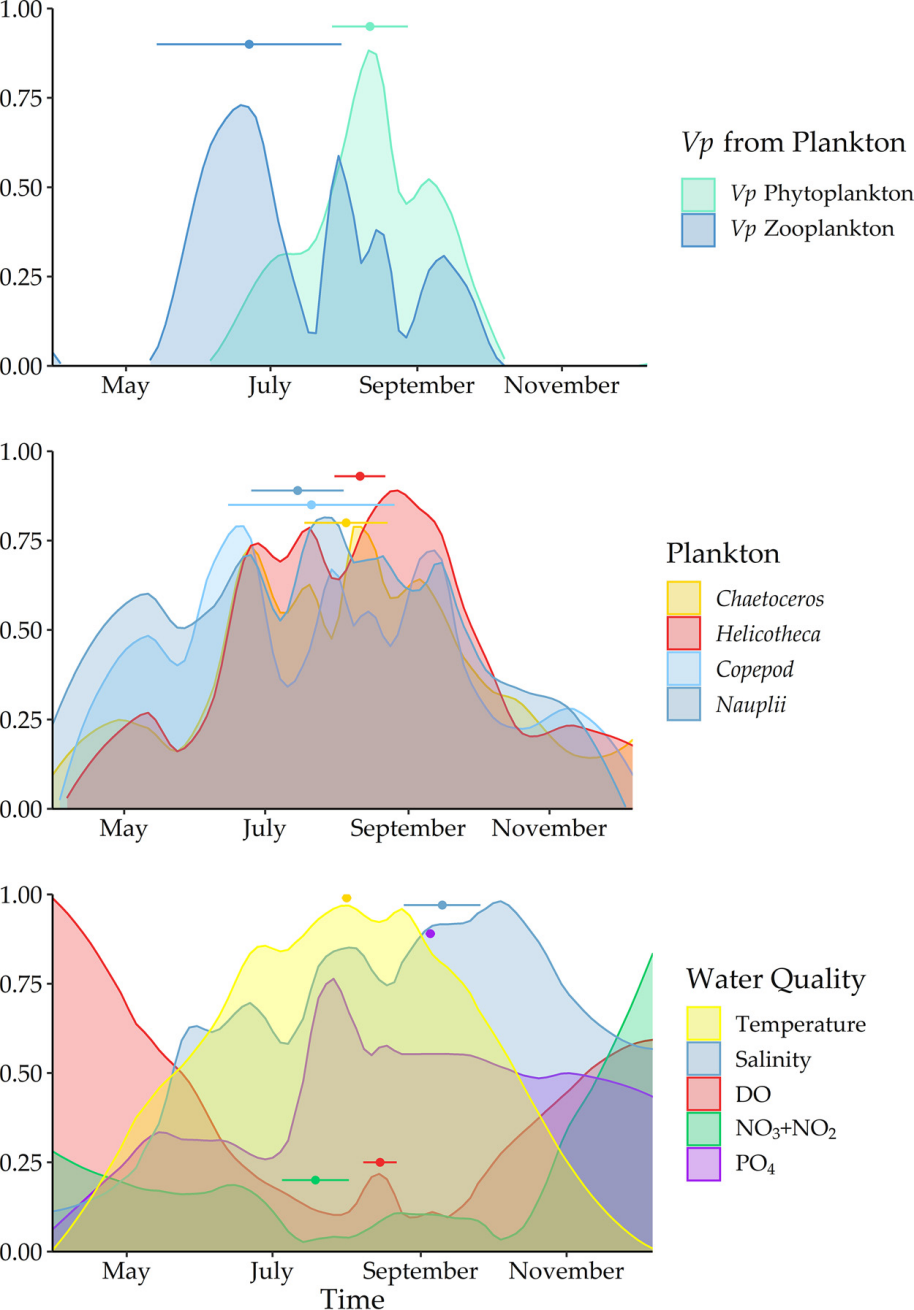
The global maxima (values normalized from 0 to 1) of the highly seasonal variables illustrated by the peak timing of (top) V. parahaemolyticus concentrations in phyto- and zooplankton, (middle) zoo and phytoplankton, and (bottom) environmental variables superimposed by year. The nadir (where variables reach absolute minima rather than maxima) is shown for NO_3_ + NO_2_ and PO_4_. Peak timing or nadir is indicated by a point and CI is represented by a bar for each variable.

V. parahaemolyticus concentrations from plankton were bimodal (Fig. 5). V. parahaemolyticus phytoplankton were temporally similar to and correlated with *Chaetoceros* spp., and *Helicotheca tamensis*. V. parahaemolyticus from zooplankton was strongly correlated and seasonally similar to the individual components of nauplii and copepods. In early spring and late fall, *Chaetoceros* spp., *Helicotheca tamensis*, nauplii, and copepods were detected in the water column, but V. parahaemolyticus was not detected in phyto- and zooplankton. The seasonal water quality will be important for understanding these bimodal patterns and differential detection. Both V. parahaemolyticus are statistically associated with water temperature. However, V. parahaemolyticus concentrations in zooplankton (peak timing at day 188 ± 34) were highest prior to the warmest water temperatures, and the highest concentrations of V. parahaemolyticus from phytoplankton (peak timing at day 224 ± 16) occurred approximately one week after the peak timing of water temperature.

NO_3_ + NO_2_, DO, and photoperiod were most strongly related to nauplii and V. parahaemolyticus concentrations from zooplankton in both correlative relationship and seasonal timing. Salinity and PO_4_ were most strongly correlated with V. parahaemolyticus concentrations from phytoplankton and individual phytoplankton taxa. These environmental variables covaried positively with water temperature, unlike DO and NO_3_ + NO_2_, though their maxima occurred approximately a month after the maxima of water temperature and were similar in timing to the individual phytoplankton taxa and V. parahaemolyticus from phytoplankton.

## DISCUSSION

V. parahaemolyticus concentrations cultured from plankton in the GBE are highly seasonal, are bimodal, and are most closely associated with individual plankton taxa rather than total plankton abundance. V. parahaemolyticus concentrations from phyto- and zooplankton are differentially associated with water temperature, salinity, pH, DO, NO_3_ + NO_2_, and PO_4_, which suggests niche-specific preferences. Importantly, common water quality variables and indicators of plankton abundance or V. parahaemolyticus associated with plankton, such as TDN and chlorophyll *a*, were not statistically or temporally associated with the dynamics of either group, which provides important insight into why these variables did not contribute well to previous V. parahaemolyticus modeling efforts (8) in this region. Very few recent data on plankton communities and their dynamics in the GBE were available prior to this study, so the results of this work provide an important update for this area, and this study can be built on for continued long-term, robust assessment of plankton-V. parahaemolyticus dynamics and monitoring in this region.

V. parahaemolyticus concentrations were most closely associated with a subset of plankton taxa and this interaction appears to depend heavily on the prevailing water quality conditions. In general, the presence or concentration of plankton overall, nutrients, or chlorophyll *a* was not a reliable indicator that V. parahaemolyticus was present at detectable levels. V. parahaemolyticus from plankton was detected only in warm weather months, whereas phytoplankton taxa were present in all samples between March to December. Notably, peak plankton-associated V. parahaemolyticus concentrations (0.018 to 21 cells/liter) did not correspond with peak plankton concentrations (10,000 to 35,000 cells/liter), regardless of the taxa. Similarly, V. parahaemolyticus from zooplankton was intermittent and at low abundance, and often V. parahaemolyticus was not cultured from zooplankton samples, even though zooplankton taxa were present in the water column.

The majority of plankton taxa observed throughout this study were diatoms, which is consistent with previous reports on the GBE ecosystem (23, 24). Only 7 samples exceeded 10,000 cells/liter, which could be considered a low level of productivity relative to other regions that frequently experience concentrations exceeding 10^6^ cells/liter (25, 26). Three taxa were the primary drivers for each of the seven highest-concentration samples: *Chaetoceros* spp. (*n* = 5), *Navicula* spp. (*n* = 1), and *Skeletonema* spp. (*n* = 1). *Skeletonema* spp. were detected in the cooler spring months in the GBE, and elevated concentrations of *Skeletonema* spp. were detected when sampling could begin safely in early March or April. The single midsummer “bloom” of *Navicula* spp. in 2015 was unexpected, as *Navicula* spp. were generally detected year-round at low levels. *Chaetoceros* spp. dominated most warm-water samples, and their abundance was statistically associated with *Helicotheca tamensis*.

The taxa that made up the plankton community in this study have been reported around the world from a broad range of environmental conditions. It is also interesting that *H. tamensis* was absent in prior analyses of the plankton community of the GBE (23, 24). The global distribution of these taxa indicates that they are capable of adaptation to a wide range of environmental conditions (27–30). Since these taxa display such a wide-ranging geographic and ecological distribution, we suggest due caution and consideration before inferring ecological insights or drawing parallels between growing regions based on taxonomic identification.

The pronounced seasonality of climatic conditions in the Northeast United States presents unique analytic challenges (8). In this temperate region, water temperature is the dominant seasonally driven variable, and so the majority of seasonal environmental variables are highly intercorrelated and related to water temperature (31–33). For this reason, seasonality models using harmonic regression or photoperiod and peak timing were used to overcome this intercorrelation to consider the individual correlative relationships and temporal dynamics of the individual variables.

Harmonic regression analysis enabled the identification of key seasonal features. For example, the highest concentrations of V. parahaemolyticus from phytoplankton occurred just after the warmest water temperatures in late August and early September (day 224 ± 16) and were most correlated with and temporally similar to patterns in water temperature, salinity, PO_4_, and *H. tamensis*. V. parahaemolyticus concentrations from zooplankton, however, were highest earlier in the year around mid-June to early July (day 188 ± 34), preceding the warmest water conditions and at approximately the time when nauplii were most concentrated in the water column. Nauplii and V. parahaemolyticus from zooplankton were also positively correlated and temporally associated with photoperiod and inversely with DO, pH, and NO_3_ + NO_2_, i.e., a different set of water quality parameters than those associated with phytoplankton. Work from other regions has also observed that V. parahaemolyticus from different matrices can have distinctly associated water quality conditions (2, 33, 34). However, this is the first study to identify the temporal and seasonally sequential dynamics of V. parahaemolyticus concentrations between matrices, which can serve as an important focus for future work.

A relationship between photoperiod and plankton community dynamics with V. parahaemolyticus concentrations was also observed by Gilbert et al. (35) and Nilsson et al. (34) in other coastal areas. They suggest that the major driver of microbial community assemblage is likely photoperiod-driven water temperature, and the fine-scale variation and turnover could be attributed to more subtle microcosm-level dynamics like shifting nutrient availability or water quality conditions. In our study, V. parahaemolyticus concentrations from zooplankton and total zooplankton, as well as NO_3_ + NO_2_, were strongly associated with photoperiod, whereas V. parahaemolyticus from phytoplankton, *H. tamensis*, water temperature, salinity, and PO_4_ and peak measurements estimated with harmonic regression occurred approximately 1 month after the longest day of the year, corresponding with earlier suggestions of unique ecological drivers for different components of the microbial community.

Nutrient availability is likely one of the most important limiting factors of plankton and V. parahaemolyticus dynamics (17, 34, 36). However, the majority of nutrient variables were not temporally or significantly correlated with V. parahaemolyticus in plankton or to individual plankton taxa in this study. Likewise, chlorophyll *a* is one of the most frequently used variables to study plankton and V. parahaemolyticus dynamics, though the strength of this association is also variable between studies (2, 32, 37).

In this study, the concentration of chlorophyll *a* was generally low (6.3 ± 4.5 μg/liter). Chlorophyll *a* was not correlated with either plankton dynamics or taxon blooms, nor was it associated temporally or statistically with V. parahaemolyticus in zoo- or phytoplankton. There is work that has shown that V. parahaemolyticus can also support a free-living lifestyle in the water column by subsisting off nutrient-rich floccules or polysaccharide exudate (35, 38). This independent lifestyle could provide insight into why V. parahaemolyticus dynamics are not related to chlorophyll *a* or observed plankton blooms in the GBE. Alternate variables to chlorophyll *a* should be considered to monitor plankton dynamics in the GBE, at least at the size fraction of >53 μm.

The dynamics between nutrients, plankton and V. parahaemolyticus are complex, and a lack of significance between variables here could relate to some dimensions of these dynamics that were not accounted for in this study, like the timing of nutrient loading events and both plankton blooms and their decline (39–43). Future work with more frequent sampling could provide an improved resolution of the contribution of nutrients to the microbial community dynamics in the GBE, but they do not appear to be directly related enough to be useful indicators of plankton-associated V. parahaemolyticus dynamics in the GBE at this temporal resolution.

### Conclusions.

We developed an in-depth ecological study to describe the plankton-V. parahaemolyticus dynamic in the GBE and assess if the plankton community could contribute to observed in changes V. parahaemolyticus, as it has been suggested for other regions around the world (3). The outcome of this work suggests that the plankton-V. parahaemolyticus dynamic in the GBE is highly seasonal, differs between phyto- and zooplankton, and is dependent on prevailing water quality conditions. Importantly, commonly used variables such as chlorophyll *a*, most nutrients and overall plankton abundance may not be informative leading indicators of plankton or V. parahaemolyticus dynamics and disease risk in this region. Though it is unclear, at this stage, how this relationship may function long-term, this preliminary study provides a basis for characterizing this complex and important ecological relationship in this region that can be integrated into long-term, ongoing surveillance to more effectively study, monitor, and manage V. parahaemolyticus disease risk for the Northeast United States.

## MATERIALS AND METHODS

### Study site and environmental sampling.

The study area was the Great Bay estuary of New Hampshire, near Nannie Island (NI), which has an important oyster (*Crassostrea virginica*) bed and is a long-term monitoring location (8, 12) (Fig. 1).

Continuous (15-min intervals [Q15]) water temperature, salinity, dissolved oxygen (DO), pH, and turbidity data were obtained from the Stormwater Management Program (SWMP) from 2014 to 2016 for times simultaneous with and preceding sampling events at a SWMP datasonde site (station GRBGB) in close proximity (43.07220, 70.86940) to the NI study site. Grab water samples were collected on each sample date and processed for water quality analyses, including nonpurgeable organic carbon (NPOC), total dissolved nitrogen (TDN), nitrate and nitrite (NO_3_ + NO_2_,) ammonium (NH_4_), orthophosphate (PO_4_), dissolved organic nitrogen (DON), total suspended solids (TSS), particulate carbon (PC), particulate nitrogen (PN), chlorophyll *a* (CHL) and pheophytin (PHEO) measurements. Data for these parameters were also obtained from the SWMP database (https://cdmo.baruch.sc.edu/dges/). Separate water temperature, salinity, pH, and dissolved oxygen (DO) measurements using YSI 6600 and EXO multiprobe sondes (Yellow Springs Instruments, Yellow Springs, OH), were collected concurrently with sampling at low tide from the NI study site.

### Plankton collection and sample processing.

Two plankton samples were collected with 53-μm mesh netting. In year 1, a student net (Aquatic Instruments, FL, USA) was used during 10 weighted tows to collect ∼140 liters of water. In year 2, a Niskin sampler with a student net was used to collect 160 liters. In year 3, a 30-liter Schindler-Patalas trap (Wildco, FL, USA) with a student net was used to collect 180 liters. All sample counts were transformed to cells per liter to standardize comparisons between years. One sample was phototactically separated using published methods and the plankton separation equipment described previously (44). The separated phyto- and zooplankton fractions were filtered in 53-μm Nitex bolting cloth (Wildco, FL, USA) filter cones. The separated and filtered fractions were air dried and weighed. The second plankton sample was filtered, resuspended to a volume of 50 ml in <53-μm filtrate water from NI, and preserved with 1% sucrose formalin (45).

### Plankton identification and enumeration.

The second plankton sample was analyzed with a phase-contrast microscope at ×400 magnification (Olympus, USA) to count 10 nonconsecutive columns (100 quadrants of the Sedgwick rafter grid). Plankton enumeration and concentration determinations were consistent with standard methods for plankton analysis (45) using a phase-contrast microscope and a 1-ml grafted Sedgwick rafter (Wildco, FL, USA). Phytoplankton were identified to the genus or species level and zooplankton to higher taxa or functional groups. Plankton identification was confirmed with resources from the work of Dolan and Cooper and Baker et al. (46, 47). Sample dilution was conducted, when necessary, for samples that were too abundant for accurate counting using deionized (DI) water.

### V. parahaemolyticus concentration enumeration.

Phyto- and zooplankton samples were analyzed for associated V. parahaemolyticus concentrations according to previously published methods with a 3-tube MPN alkaline peptone water enrichment and *Vibrio* CHROMagar (48). Probable V. parahaemolyticus isolates were confirmed by PCR detection of the *tlh* gene (6, 8, 48). V. parahaemolyticus concentrations associated with phyto- and zooplankton were transformed from MPN per gram to MPN per liter. Samples without detected V. parahaemolyticus were determined to be below the limit of detection and were assigned a standard value of <0.03.

### Statistical analysis.

All statistical analyses were performed in the R Statistical Program and Environment, version 3.5.3 (49), and the vegan Community Ecology Package, version 2.5-2 (50). MPN values for V. parahaemolyticus concentrations were log transformed and plankton counts were log+1 transformed to approximate normality and reduce skewness. Significance for all analyses was determined by a *P* value of <0.05.

### Plankton abundance analysis.

Plankton community total abundance (TA), relative abundance (RA), species richness, evenness, and Shannon H diversity were calculated for the study. The taxa observed in collected samples were classified as abundant (>4% of sample total abundance), common (≤4% and ≥0.1%), or rare (<0.1%) in the GBE plankton community. Rare taxa (abundance < 0.1%) were not included in univariate, multivariate, or seasonality analysis.

### Seasonality.

Seasonal community assemblage was assessed between calendar seasons (spring, March to 21 June; summer, 22 June through 21 September; and fall, 22 September to December) and years (2014, 2015, and 2016) by permutational multivariate analysis of variance (PERMANOVA), multiresponse permutation procedure (MRPP), and indicator species analysis (ISA). The seasonality and general trend for all variables were also explored with models 1 and 2:

(1) $$E\left(Y_{t}\right)={\text{β}}_{0}+{\text{β}}_{1}t+{\text{β}}_{p}\text{photoperiod}$$

(2) E(Yt)=β0+β1t +βs sin⁡(2πωt)+βc cos⁡(2πωt)

to fit periodicity and trends of the seasonal oscillations (6). In both models, $Y_{t}$ is the daily time series for the outcome of interest, β_0_ is the intercept, *t* is the daily time series, and β_1_ indicates a general trend in the variable of interest. Model 1 contains the photoperiod variable β*_p_*photoperiod, and model 2 uses harmonic regression terms for the calendar day in the study where β*_s_* and β*_c_* are the coefficients of the harmonic terms and ω is the term representing the annual cycle (365.25 days, ω = 1/365.25). The peak timing of the periodic oscillations identified by model 2 was determined by calculating the phase shift:

(3) $$\text{ψ}=\text{arctan}\left(\frac{{\hat{\beta }}_{S}}{{\hat{\beta }}_{c}}\right)+k$$

When ${\hat{\beta }}_{S}$ and ${\hat{\beta }}_{c}$ were positive, *k* was 0. When ${\hat{\beta }}_{S}$ was <0 and ${\hat{\beta }}_{c}$ was >0, *k* was equal to 2π, and when ${\hat{\beta }}_{S}$ and ${\hat{\beta }}_{c}$ were negative, or when ${\hat{\beta }}_{S}$ was >0 and ${\hat{\beta }}_{c}$ was <0, then *k* was equal to π. The phase shift (ψ) was then multiplied by 365.25 to account for leap years. Confidence intervals (CI) of the peak timing were calculated by determining the estimated variance of the phase shift (ψ), where ${\text{σ}}_{{\text{β}}_{c}{\text{β}}_{s}}$ is the covariance and ${\hat{\beta }}_{s}^{2}$ and ${\hat{\beta }}_{c}^{2}$ are the variances of ${\text{β}}_{s}$ and ${\text{β}}_{c}$ in the following equation:

(4) $$\text{var}\left(\text{ψ}\right)=\frac{{\left({\text{σ}}_{\text{β}s}{\text{β}}_{c}\right)}^{2}+{\left({\text{σ}}_{{\text{β}}_{c}}{\text{β}}_{s}\right)}^{2}-(2{\text{σ}}_{{\text{β}}_{c}{\text{β}}_{s}}{\text{β}}_{s}{\text{β}}_{c})}{({\hat{\beta }}_{s}^{2}+{\hat{\beta }}_{c}^{2}){\left({\hat{\beta }}_{s}^{2}+{\hat{\beta }}_{c}^{2}\right)}^{2}}$$

CI values for the peak timing estimates were determined as $1.96\times \sqrt{\text{var}\left(\text{ψ}\right)\times 365.25/2\text{π}}$. The seasonality of the environmental variables was evaluated by the significance of the coefficients, the deviance explained, Akaike information criterion (AIC), and coefficient of determination (*r*^2^) value of models 1 and 2 (51, 52).

### Correlation.

Spearman rank correlation analyses were performed for all variables to assess the relationship of environmental variables to V. parahaemolyticus dynamics in plankton.
