# Supplementary material for: The Seasonal Microbial Ecology of Plankton and Plankton-Associated Vibrio parahaemolyticus in the Northeast United States
Source: Appl Environ Microbiol. 2021 Jul 13;87(15):e02973-20. doi: 10.1128/AEM.02973-20 (PMC8276809; doi:10.1128/AEM.02973-20)
Supplement: Supplemental file 1 — Tables S1 and S2, Fig. S1 and S2. Download AEM.02973-20-s0001.pdf, PDF file, 1.98 MB [file aem.02973-20-s0001.pdf]

Table S1. Range and mean values for *V. parahaemolyticus (Vp)*, water quality and nutrients by year.

Variable	Period	Min	Max	Mean and SD
Vp and Plankton				
Phytoplankton *Vp* (MPN/L)	2014-2016	0.018	21	1.7 ± 4
	2014	0.018	21	4.6 ± 7.5
	2015	0.018	10.8	1.8 ± 3.4
	2016	0.018	11.7	3.2 ± 1.5
Zooplankton *Vp* (MPN/L)	2014-2016	0.018	14	8.5 ± 2.6
	2014	0.018	4.1	1.6 ± 1.5
	2015	0.018	1	0.2 ± 0.3
	2016	0.018	14.3	1.1 ± 3.7
Phytoplankton Abundance (n/L)	2014-2016	62	35,630	6474 ± 10,980
	2014	62	35,598	9,038 ± 15,988
	2015	119	31,775	6726 ± 10,055
	2016	89	35,630	4986 ± 9,124
Zooplankton Abundance (n/L)	2014-2016	0	3,350	227 ± 381
	2014	7	308	113
	2015	6	3,350	445
	2016	3	322	107
Water Quality				
DON (mg/L)	2014-2016	0	0.2	0.12 ± 0.05
	2014	0.13	0.19	0.16 ± 0.02
	2015	0.01	0.19	0.14 ± 0.05
	2016	0	0.15	0.11 ± 0.04
NH_4 (mgN/L)	2014-2016	0	0.1	0.02 ± 0.02
	2014	0.01	0.04	0.02 ± 0.01
	2015	0	0.04	0.01 ± 0.01
	2016	0	0.07	0.03 ± 0.02
$NO_3 + NO_2$ (mgN/L)	2014-2016	0.01	0.2	0.04 ± 0.04
	2014	0.01	0.09	0.03 ± 0.04
	2015	0.01	0.1	0.04 ± 0.03
	2016	0.01	0.2	0.05 ± 0.05
NPOC (mg/L)	2014-2016	0.36	3.8	2.31 ± 0.7
	2014	1.85	3.22	2.6 ± 0.6
	2015	0.36	3.14	2.4 ± 0.7
	2016	1.07	3.8	2.2 ± 0.7
PC (mg/L)	2014-2016	0.43	3.6	1.16 ± 0.7
	2014	0.43	1.73	1.14 ± 0.6
	2015	0.44	3.63	1.23 ± 0.9
	2016	0.49	2.77	1.11 ± 0.6
PN (mg/L)	2014-2016	0.05	0.5	0.17 ± 0.1

	2014	0.06	0.24	0.15 ± 0.01
	2015	0.05	0.44	0.18 ± 0.12
	2016	0.06	0.49	0.17 ± 0.12
PO_4 (mgP/L)	2014-2016	0	0.07	0.03 ± 0.02
	2014	0.01	0.04	0.03 ± 0.01
	2015	0.01	0.07	0.03 ± 0.02
	2016	0	0.05	0.03 ± 0.02
TDN (mg/L)	2014-2016	0.06	0.34	0.18 ± 0.06
	2014	0.16	0.26	0.21 ± 0.04
	2015	0.06	0.28	0.19 ± 0.05
	2016	0.07	0.34	0.17 ± 0.07
Chlorophyll-*a* (µg/L)	2014-2016	1.3	22.6	6.3 ± 4.5
	2014	2.04	8.38	5.9 ± 2.4
	2015	2.26	22.55	7.1 ± 6.1
	2016	1.31	14.48	5.9 ± 3.8
Dissolved Oxygen (mg/L)	2014-2016	6.5	11.5	8.5 ± 1.3
	2014	6.24	12.31	8.5 ± 1.2
	2015	6.6	11.31	8.4 ± 1.1
	2016	6.36	12.74	8.5 ± 1.4
pH	2014-2016	7.5	8	7.8 ± 0.15
	2014	7.37	8.35	7.8 ± 0.19
	2015	7.4	8.06	7.8 ± 0.15
	2016	7.62	8.2	7.9 ± 0.11
Pheophytin (µg/L)	2014-2016	0.7	9.8	2.9 ± 2.2
	2014	1.58	5	2.8 ± 1.4
	2015	0.66	9.83	3.5 ± 2.9
	2016	0.71	7.76	2.6 ± 1.8
Salinity (ppt)	2014-2016	14.1	32.1	27.1 ± 3.5
	2014	17.79	31.76	26.2 ± 3.3
	2015	14.11	31.29	27.1 ± 3.5
	2016	18.2	32.09	27.9 ± 3.5
Water temperature (°C)	2014-2016	6.3	25.3	17.7± 5.1
	2014	3.52	26.97	15.3± 5.5
	2015	5.79	23.48	16.2± 5.3
	2016	5.39	24.04	15.6± 5.5
Total suspended solids	2014-2016	10.7	76.4	29.8 ± 15.6
	2014	10.71	33.57	19.2 ± 10.9
	2015	13.33	76.43	29.6 ± 17.6
	2016	11.06	69.76	32 ± 15
Turbidity (NTU)	2014-2016	1.15	16.4	10.8 ± 13.3
	2014	0.92	2.73	1.6 ± 0.7
	2015	1.71	3.8	2.5 ± 0.8

	2016	1.51	16.4	2.2 ± 0.5

Figure S1. *V. parahaemolyticus*, plankton species and environmental variables by time with peak timing and confidence intervals.

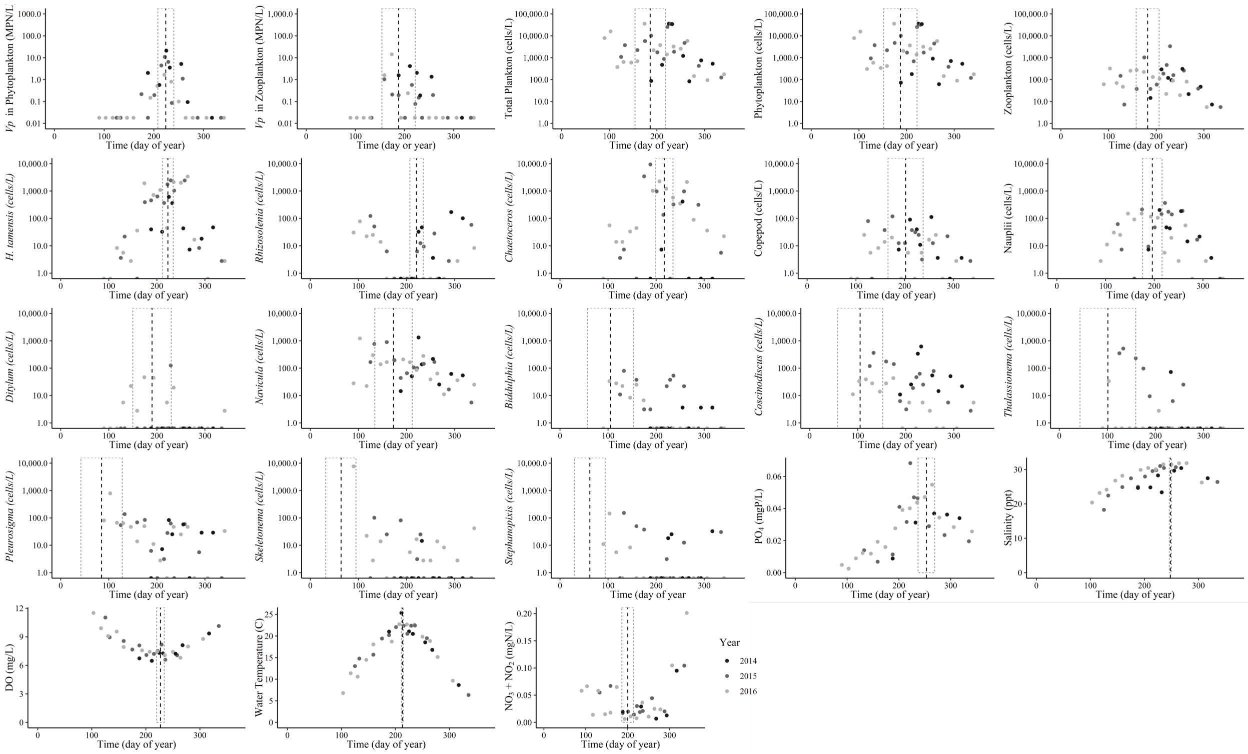

Figure S2. Environmental variables with limited seasonal periodicity.

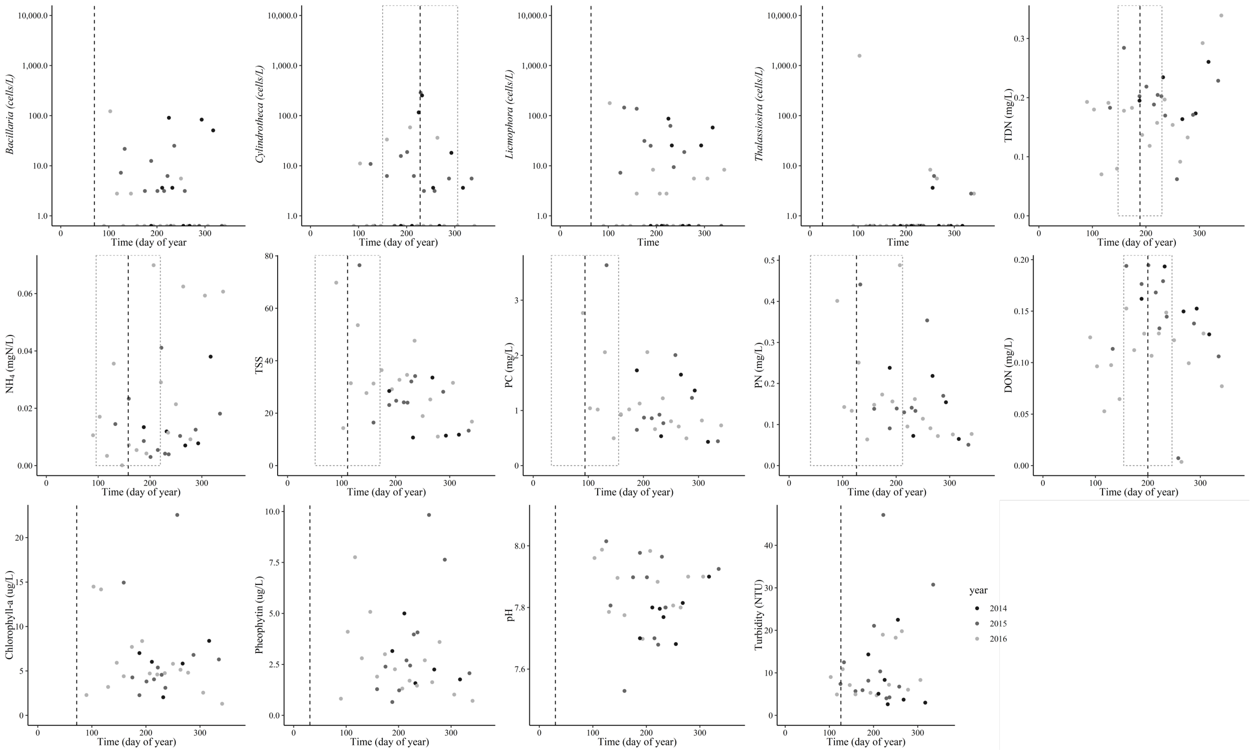

Table S2. Environmental variables showing little evidence of seasonality. Model 1 (top) and Model 2 (bottom)

	Coefficient		Standard Error		r^2	Deviance Explained	AIC	Peak Timing
	trend	seasonality	trend	seasonality				
Bacillaria	-0.001*	0.02	<0.001	0.13	0.08	0.13	129.26	
	-0.002*	0.47 0.19	<0.001	0.36 0.44	0.1	0.18	129.36	70 ± 88
Cylindrotheca	-0.002	0.05	0.001	0.17	0.02	0.07	146.57	
	-0.001	-0.45 -0.45	0.001	0.46 0.56	0.02	0.1	147.31	228 ± 78
Licmophora	-0.001	0.02	0.001	0.17	0.01	0.04	149.67	
	-0.002	0.52 0.26	0.001	0.48 0.59	0.01	0.08	150.39	64 ± 102
Thalassiosira	<0.001	-0.15	0.001	0.13	0.02	0.07	128.04	
	<0.001	0.34 0.70	0.001	0.36 0.44	0.02	0.11	128.55	26 ± 47
Tintinnida	<0.001	0.45	0.001	0.2	0.08	0.13	160.55	
	<0.001	0.57 -1.21	0.001	0.57 0.70	0.067	0.14	161.95	157 ± 56
DON	<0.001**	<0.01	<0.001	<0.001	0.26	0.31	104.87	
	<0.001***	-0.008; -0.03*	<0.001	0.01 0.01	0.27	0.34	102.12	200 ± 45
NPOC	-0.001	-0.02	<0.001	0.07	0.03	0.10	73.3	

	-0.001	0.32 0.23	<0.001	0.20 0.23	0.09	0.18	72.24	55 ± 56
TDN	<0.001	-0.01 <0.001	<0.001	<0.001 <0.001	0.11	0.17	82.39	
	<0.001	<0.001**	<0.001	<0.001	0.09	0.18	81.07	189 ± 41
NH4	<0.001	<0.001* <0.001	<0.001	<0.001 <0.001	0.20	0.24	156.49	
	<0.001*	<0.001	<0.001	<0.001	0.16	0.24	15.82	158 ± 62
TSS	0.008	2.46	0.01	1.51	0.03	0.10	253.48	
	0.005	9.22 -3.31	0.01	4.22 4.89	0.12	0.21	251.54	111 ± 60
PC	-0.0003	0.08	0.0005	0.07	0.006	0.06	71.61	
	-0.0005	0.44 -0.02	0.0005	0.20 0.24	0.09	0.19	69.19	94 ± 61
PN	<0.001	0.02 <0.001	<0.001	0.001 <0.001	0.02	0.08	40.66	
	<0.001	<0.001	<0.001	<0.001	0.02	0.13	40.08	126 ± 86
DON	<0.001***	<0.001 <0.001	<0.001	<0.001 <0.001	0.20	0.31	104.87	
	<0.001	<0.001	<0.001	<0.001	0.26	0.33	103.93	200 ± 46
CHL	-0.001	0.08	0.003	0.45	0.06	0.01	193.56	
	-0.002	1.44 0.48	0.003	1.37 1.53	0.06	0.04	194.43	72 ± 107
Pheophytin	<0.001	-0.02	0.001	0.22	0.06	0.01	146.72	
	<0.001	0.06 0.11	0.002	0.67 0.75	0.09	0.01	148.7	31 ± 200
pH	<0.001	-0.01 <0.001	<0.001	0.01 <0.001	0.05	0.11	46.66	
	<0.001	<0.001	<0.001	<0.001	0.10	0.19	47.64	30 ± 36
Turbidity	<0.001	-0.93	0.006	1.03	0.04	0.03	247.21	
	0.001	-2.27 1.39	0.006	2.80 3.70	0.06	0.04	248.83	126 ± 166

significance of coefficients indicated as *** 0.001, ** 0.01, * 0.1
